# Involvement of the European Medicines Agency in multi-stakeholder regulatory science research projects: experiences of staff members and project coordinators

**DOI:** 10.3389/fmed.2023.1181702

**Published:** 2023-05-30

**Authors:** Robbe Saesen, Matilde Machado, Bianca Crifo, Lifang Liu, Corinne de Vries, Ralf Herold, Jordi Llinares Garcia, Isabelle Huys

**Affiliations:** ^1^Clinical Pharmacology and Pharmacotherapy Research Unit, Department of Pharmaceutical and Pharmacological Sciences, KU Leuven, Leuven, Belgium; ^2^European Organisation for Research and Treatment of Cancer (EORTC), Brussels, Belgium; ^3^Faculty of Health, Medicine and Life Sciences, Maastricht University, Maastricht, Netherlands; ^4^Independent Researcher, Milan, Italy; ^5^Translational Sciences Office, European Medicines Agency, Amsterdam, Netherlands; ^6^Task Force Regulatory Science and Innovation, European Medicines Agency, Amsterdam, Netherlands

**Keywords:** academia, regulatory science, Europe, Innovative Medicines Initiative, Horizon 2020, Marie Skłodowska-Curie Actions, interviews, qualitative research

## Abstract

**Background:**

The European Medicines Agency (EMA) interacts with many different stakeholders involved in the development of drugs, including academic researchers. In recent years, EMA has collaborated more closely with academia, *inter alia* by taking part in external research projects such as those set up under the Horizon 2020 program in general and the Innovative Medicines Initiative in particular. The aim of this study was to evaluate the perceived added value of EMA’s involvement in these projects, both from the perspective of the Agency’s participating Scientific Officers and of the coordinators of the consortia that undertook them.

**Methods:**

Semi-structured interviews were conducted with the coordinators of 21 ongoing or recently finalized projects in which EMA has participated, as well as with the Agency experts contributing to them.

**Results:**

In total, 40 individuals were interviewed, of whom 23 were project coordinators and 17 were EMA staff members. While most of the projects were reported to suffer from delays due to the SARS-CoV-2 pandemic, the consortia adapted to the circumstances and their members still expected to deliver on their objectives. EMA’s input into the projects ranged from providing guidance by reviewing documents and attending meetings to creating project materials and disseminating them. The frequency of communication between EMA and the consortia varied widely. The projects generated a diverse set of outputs, which encompassed new or improved medicinal products, methodological standards, research infrastructures, and educational tools. All of the coordinators expressed that EMA’s contributions to their projects had increased the scientific relevance of their consortium’s work, and the EMA experts found that the knowledge and the deliverables produced by the projects were valuable, taking into consideration the time they had invested into them. In addition, interviewees highlighted some actions which could be taken to increase the regulatory significance of the project outcomes.

**Conclusion:**

EMA’s engagement in external research projects benefits the consortia conducting them and supports the Agency’s mission to foster scientific excellence and advance regulatory science.

## 1. Introduction

The European Medicines Agency (EMA) is a decentralized body of the European Union (EU) headquartered in Amsterdam, Netherlands (1). Its core responsibility is the protection and promotion of public and animal health through scientific excellence in the evaluation and the supervision of medicines for human and veterinary use (1). The Agency’s main activities include (a) supporting the development of medicinal products and enabling timely patient access to them, (b) assessing marketing authorization applications submitted through the so-called centralized procedure, (c) monitoring the safety of medicinal products, both before and after they are available on the market, and (d) making clear and objective information about medicines available to patients and healthcare professionals (1).

To deliver on its mission, EMA closely coordinates with a network of experts from the national competent authorities (NCAs) of the Member States of the European Economic Area (EEA; i.e., the Member States of the EU plus Norway, Iceland, and Liechtenstein) (1). These experts are members of EMA’s scientific committees (e.g., the Committee for Human Medicinal Products, CHMP), working parties (e.g., the Scientific Advice Working Party, SAWP) or other groups, and as such contribute to the Agency’s work (1, 2). Together, EMA, the NCAs and the European Commission form the European Medicines Regulatory Network (EMRN), which safeguards the efficacy, the safety and the quality of drugs that are available on the EU market and addresses the challenges and improvements needed in this respect (1).

Beyond the EMRN, EMA also systematically engages with other stakeholders in the medicines development process, including the pharmaceutical industry, patients and consumers, healthcare professionals, and health technology assessment (HTA) bodies (3).

Furthermore, to achieve the goals listed in the EMA Regulatory Science to 2025 (4) and the European medicines agencies network strategy to 2025 (5) reflection documents, the Agency has in recent years been increasingly engaging with academia (including universities, learned societies, not-for-profit organizations, research consortia, etc.). In this regard, EMA has established an academia liaison office and created a framework for collaboration with academia (6) as well as an action plan for its internal Academia Collaboration Matrix (7). The rationale behind this progressive intensification of the relationship between EMA and academia is fourfold (6):

• To increase understanding of EMA’s public health role among academic stakeholders, thereby increasing the trust of academia in the regulatory system.
• To facilitate the translation of academic research into novel methodologies and medicinal products which meet EMA’s standards and address public and animal health needs.
• To ensure that EMA can depend on the expertise of its academic partners to inform its decision-making.
• To collaborate on advancing the field of regulatory science^1^ by fostering the development of new biomarkers, endpoints, and methodologies.

As part of its efforts to cooperate more closely with academia, EMA contributes to several external research projects that are coordinated by academic institutions and that address issues which are of relevance to the field of regulatory science (8). These projects have mainly been undertaken by large consortia comprised of public and private stakeholders, working together to achieve common objectives in the pre-competitive space of research and development, not seeking to develop any commercial or medicinal products. In most cases, they have been set up within the context of the EU Horizon 2020 (H2020) framework (9). While some have been exclusively financed with public funds [including multiple Coordination and Support Actions and Marie Skłodowska-Curie Actions, abbreviated as CSAs and MSCAs, respectively (10)], others have also been initiated with in-kind contributions from the pharmaceutical industry, as part of the Innovative Medicines Initiative (IMI) public–private partnership (11). EMA’s participation in such projects is usually triggered by a specific request from the consortium members.

The criteria employed by the Agency to decide on whether or not to participate in a particular project revolve around the project’s relevance for EMA’s strategic aims, the anticipated added value that EMA can bring to the project, the project’s potential for reinforcing international and inter-institutional partnerships between EMA and other organizations, and practical considerations (e.g., the quality of the research proposal, the resources required, the risk of conflicts of interest^2^ in light of the Agency’s statutory obligations, etc.) (8). For projects featuring companies as partners, the Agency will not get involved if the project includes any activities to develop a commercial or medicinal product. EMA is open to being approached early on, but only commits to engaging with consortia when they are no longer in the competitive stages of their grant applications, in compliance with its policy to maintain a level playing field amongst applicants (8).

The involvement of EMA in externally funded regulatory science projects can take different forms, varying in terms of the roles and responsibilities assumed by the Agency’s expert(s) involved (8). More specifically, EMA can participate as:

• A member of the advisory board, the steering committee or an equivalent body of the project, which implies that the Agency’s expert(s) provide strategic input on the project without EMA being a formal member of the consortium. This role involves limited time and resource commitments and does not allow the Agency to influence the initial research plan.
• A consortium partner, which signifies that EMA shares responsibility for producing the deliverables specified in the project agreement as a task member, task leader or work package leader. This role involves considerable time and resource commitments but allows the Agency to shape the initial research plan.
• A project coordinator, which means that EMA exceptionally takes the lead in setting up and managing the project. This role involves substantial time and resource commitments and allows the Agency to compose the initial research plan.

Regardless of whether the Agency chooses to accept an offer to take part in a project, the consortium behind the project is strongly recommended to make use of the various regulatory tools and procedures that EMA has available to support the development of innovative medicines and methodologies (12), including scientific advice (13), qualification advice and opinions (14), and the services provided by the Innovation Task Force (ITF) (15).

While the scientific and the socio-economic impact of H2020 and IMI projects have been examined in prior analyses (16–19), no evaluation has been performed so far of the added value that EMA’s involvement brings to such projects and to the work of the Agency itself. In this study, we set out to assess perceptions of EMA’s participation in externally funded regulatory science research projects, both from the perspective of the projects’ coordinators and of the EMA experts involved. Based on interviews with these stakeholders, we also aimed to formulate preliminary recommendations for an improved engagement of the Agency in future projects of this nature which would improve the chances of their outputs being translated into regulatory innovations.

## 2. Materials and methods

We searched EMA internal documentation systems to compile a list of all externally funded research projects that the Agency has been involved in since its inception. From this list, we selected the projects that either had been finalized in the 12 months preceding the start of our study or were ongoing but had been initiated more than one year before the study began in April 2021. These selection criteria allowed us to manually compose a non-random sample of projects that were at different stages of advancement with respect to their planned deliverables.

Information on these projects was then extracted from publicly accessible sources [e.g., journal articles, project websites, progress reports, IMI factsheets, the European Commission’s Community Research and Development Information Service (CORDIS), etc.] as well as from unpublished documents (e.g., research proposals, meeting notes, etc.). This information enabled us to acquire an in-depth understanding of the objectives, the structure and the governance of each project.

Next, we conducted semi-structured interviews with members of two stakeholder groups, namely, the coordinators of the projects^3^ and the EMA staff members who served on their advisory boards or contributed to one or more of their work packages. Typically, the former were experienced academic researchers and the latter regulatory scientists with strong expertise in the subject matter. Individuals targeted for recruitment were contacted by e-mail and if they were willing to be interviewed, a virtual meeting was scheduled. If a project coordinator could not be reached or declined to participate, a colleague of theirs who assisted them during the project was recruited instead. Participants provided their written informed consent prior to the interview and were sent the questions in advance. To ensure that inter-group comparisons could be made and to limit the risk of order effects bias, the project coordinators and the EMA experts received similar questions displayed in the same order. Nevertheless, some of the questions were exclusive to either stakeholder group.

The interviews were carried out by one person (RS), which negated the potential detrimental effects of interviewer variance. This person was not employed by EMA. Although interview guides were prepared (Supplementary material) and the interviews therefore followed a predefined structure, some impromptu questions may have been asked to further expand on certain topics that were brought up by the interviewees. All interview sessions were held in English via Microsoft Teams between June and August of 2021 and were audio-recorded using Snagit. The recordings were pseudonymized and subsequently transcribed *ad verbum* by either a member of the research team (RS, MM, and BC) or a third-party company. None of the transcribers were EMA staff members. The transcripts were analyzed by one researcher (RS) based on the framework method (20, 21), which comprises seven distinct steps that are completed successively (Supplementary Table 1). The analysis was performed with the help of the NVivo software.

In this manuscript, the study results are described in an aggregated manner, and quotes from the interviewees are provided for illustrative purposes. Since the sample size was small and projects were diverse in many aspects, no attempts were made to analyze the interview statements in relation to the type or duration of the project, the technical or therapeutic area of the project, professional background of the interviewees or other factors that could be considered for further interpretation.

The study was reviewed and approved by the Ethics Committee Research UZ/KU Leuven (S65593).

## 3. Results

### 3.1. Characteristics of the projects and breakdown of the interview sample

In total, 21 projects were included in this study (Table 1), of which 11 (52%) were set up under the IMI umbrella, 7 (33%) were undertaken as non-IMI H2020 actions (e.g., CSAs and MSCAs), and 3 (14%) were funded through other means (e.g., with financial support from national governments).

**TABLE 1:** Overview of the projects included in this study.

Name of project	Main objective^•^	Start date	End date	Funding mechanism	EMA role	Coordinating institution^▲^	References
AVANT	Developing and testing the efficacy and sustainability of alternatives to antimicrobials for the management of pig enteritis	January 2020	December 2024	H2020 Innovation Action	Advisory board member	University of Copenhagen	(38, 39)
ConcePTION	Creating a trusted biomedical ecosystem capable of providing evidence-based information on the safety of medications during pregnancy and breastfeeding in an efficient, systematic and ethically responsible way	April 2019	March 2024	H2020 IMI2	Consortium partner	University Medical Center Utrecht	(40, 41)
c4c	Creating a sustainable, integrated pan-European collaborative pediatric network that will speed up and facilitate the running of high-quality clinical trials in children while ensuring that the voices of young patients and their families are heard	May 2018	April 2024	H2020 IMI2	Advisory board member	Penta Foundation	(42, 43)
ECRAID-Plan	Developing the detailed business plan for a coordinated, permanent, pan-European infrastructure for clinical research on infectious diseases, which will generate rigorous evidence to improve the diagnosis, prevention and treatment of infectious diseases, and to better respond to infectious disease threats	January 2019	June 2021	H2020 CSA	Advisory board member	University Medical Center Utrecht	(44, 45)
EHDEN	Building a large-scale, federated network of European healthcare data sources standardized to a common data model, which will allow access to the health data of 100 million EU citizens	November 2018	April 2024	H2020 IMI2	Advisory board member	Erasmus University Medical Center	(46, 47)
EU-PEARL	Creating a sustainable and replicable framework that will produce a systematic approach to patient-centric trial platforms which allow multiple companies to test their candidate drugs simultaneously against a shared placebo group	November 2019	April 2023	H2020 IMI2	Advisory board member	Vall d’Hebron Research Institute	(48, 49)
EJP RD	Developing an effective rare diseases research ecosystem which improves the integration, effectiveness, and social impact of rare diseases research by developing, demonstrating, and promoting European/worldwide research, sharing clinical data, resources, procedures, knowledge and expertise, and establishing an efficient financial support model for all rare diseases research types	January 2019	December 2023	H2020 EJP Cofund	Policy board member	Inserm	(50, 51)
FLUCOP	Delivering a toolbox of standardized, validated serological assays for human influenza vaccines, enabling (a) the evaluation of a new vaccine’s ability to stimulate the immune system, and (b) the comparison of results from different laboratories	March 2015	February 2022	FP7 IMI1	Consortium partner	University of Siena Sclavo Vaccines Association	(52, 53)
Liver Forum	Advancing the regulatory sciences for the treatment of non-alcoholic fatty liver disease/non-alcoholic steatohepatitis and liver fibrosis by providing an independent and neutral venue for ongoing multi-stakeholder dialogue on issues of common interest and concern	November 2014	Indefinite	Public–private partnership between US federal government and pharmaceutical industry	Steering group member	Forum for Collaborative Research	(54)
MINDED	Advancing the diagnosis, imaging and treatment of neurodevelopmental disorders by developing a highly interdisciplinary post-doctoral training program which integrates nanomedicine, neuroscience and cognitive neuroscience robotics	January 2018	June 2024	H2020 MSCA	Hosting partner	Italian Institute of Technology	(55, 56)
PARADIGM	Creating a framework for structured, effective, meaningful, and ethical patient engagement in three key points in the medicines R&D process: research and priority setting, clinical trial design, and early dialogues with regulators and health technology assessment bodies	March 2018	November 2020	H2020 IMI2	Steering committee member	European Patients’ Forum	(57, 58)
PEARRL	Delivering novel bio-enabling formulations and new biopharmaceutical tools to predict the *in vivo* performance of these formulations by training early-stage researchers who can develop such innovations and serve as communication bridgers between research and regulatory science	May 2016	August 2020	H2020 MSCA	Hosting partner	University College Cork	(59, 60)
PERMIT	Reaching consensus and publishing recommendations on methodological standards to ensure the scientific excellence, validity, robustness, reproducibility, and acceptability of results generated by personalized medicine programs	January 2020	June 2022	H2020 CSA	Associated partner^☼^	European Clinical Research Infrastructure Network	(61, 62)
PREFER	Providing a set of systematic methodologies and recommendations to assess, engage and include patient perspectives during the development, approval, and post-approval of new therapies	October 2016	May 2022	H2020 IMI2	Advisory board member	Uppsala University	(63, 64)
PREMIER	Delivering a framework for assessing and characterizing the environmental risks of active pharmaceutical ingredients, especially older ones that have never undergone an environmental risk assessment	September 2020	August 2026	H2020 IMI2	Consortium partner	Radboud University	(65, 66)
STARS	Complementing, coordinating and harmonizing regulatory efforts among Member States and at European level to support academic health research by strengthening the dialogue between academia and regulatory authorities through earlier engagement of regulators with researchers and scientists	January 2019	June 2022	H2020 CSA	Consortium partner	Federal Institute for Drugs and Medical Devices Federal Institute for Vaccines and Biomedicines	(67, 68)
VAC4EU	Creating and implementing a European partnership that can respond rapidly and reliably to relevant questions around post-licensure vaccine coverage, benefits and risks by generating robust and trustworthy real-world evidence	October 2019	Indefinite	Self-sustained	Advisory board member	University Medical Center Utrecht	(69)
VALUE-Dx	Evaluating the medical, economic, and public health value of diagnostics in treating antimicrobial resistance by establishing the infrastructure, methods, processes, and approaches needed to generate evidence of this value	April 2019	March 2023	H2020 IMI2	Advisory board member	University of Antwerp	(70, 71)
VITAL	Providing evidence-based knowledge on vaccination strategies to establish healthy aging by mapping the burden of vaccine-preventable infectious diseases in the elderly and investigating vaccinations and immunity to infections in the aging population	January 2019	December 2023	H2020 IMI2	Advisory board member	University Medical Center Utrecht	(72, 73)
WEB-RADR 2	Expanding access to a mobile application which allows patients and healthcare professionals to report adverse drug reactions directly to the relevant authorities by making its functionalities available through application programming interfaces	September 2018	June 2020	H2020 IMI2	Advisory board member	Medicines and Healthcare Products Regulatory Agency	(74, 75)
N/A*	Assessing the effectiveness and cost-effectiveness of chimeric antigen receptor T-cell treatments for treating diffuse large B-cell lymphoma in clinical practice using real-world evidence	July 2019	February 2020	ZIN procurement procedure	Advisory board member	National Health Care Institute	N/A*

^•^The descriptions of the objectives were taken from the projects’ websites and factsheets.

^▲^Excluding commercial parties.

^☼^This role is advisory in nature.

*This project did not have a specific name and no information about it was published.

H2020, Horizon 2020; IMI, Innovative Medicines Initiative; CSA, Coordination and Support Action; EJP, European Joint Program; FP7, Seventh Framework Program; MSCA, Marie Skłodowska-Curie Action; ZIN, Zorginstituut Nederland.

**FIGURE 1:**
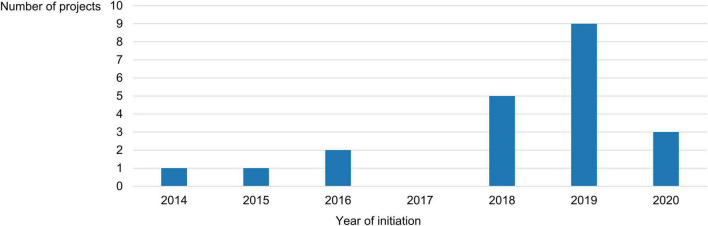
Breakdown of the projects included in this study by their year of initiation.

The role that EMA played in these projects was mainly advisory in nature (15/21, 71%), with the Agency serving as a formal partner of the consortium in 6 of them (6/21, 29%). Only 5 projects (5/21, 24%) had been finalized at the time our study ended (namely, ECRAID-Plan, PARADIGM, PEARRL, WEB-RADR 2, and an untitled project set up by the National Health Care Institute in the Netherlands); the rest (16/21, 76%) were still ongoing at that point. The longest-running project had started in 2014, while the most recently launched one had begun in 2020. More than half of all projects (12/21, 57%) had been initiated after 2018 (Figure 1). Excluding the projects that ran indefinitely (2/21, 10%), the median project had a planned or actual duration of 5 years (interquartile range: 2 years and 11 months).

The personal experience with EMA’s participation in these 21 projects was investigated through the conduct of 40 interviews. Although the aim was to recruit at least two participants for each project (one per stakeholder group), 17 EMA experts and 23 project coordinators were ultimately interviewed. This imbalance between the two target groups was caused by a number of factors: (a) some of the EMA experts were involved in multiple projects, (b) some of the projects were coordinated by two people working in tandem with each other, and (c) some of the EMA experts were unavailable and could therefore not take part in an interview. Not all of the project coordinators were academic researchers: some projects were overseen by individuals affiliated with an NCA or a patient organization.

### 3.2. Views on the status and progress of the projects and the planning of the deliverables

At the time the interviews were being conducted, the majority of the projects had not yet been completed. As an opening question, the interviewees who were taking part in an ongoing project were asked to expand on its status. The answers received reflected the diversity of the projects in terms of the progress they were making toward their objectives: some had been operational for a longer period of time and had already produced tangible outputs, while others had only been running for a year and were therefore still in their early stages, not having generated any concrete deliverables yet. Notably, the EMA experts participating in this study were not always aware of where the projects they were involved in stood exactly. This was especially observed for projects in which the Agency had an advisory role, providing input exclusively on an *ad hoc* basis. For such projects, the EMA expert is sometimes only informed of new developments when the consortium consults its stakeholders, which may only occur once or twice a year. However, the EMA experts experiencing this situation indicated that they did not require more frequent updates.

“*The progress, that has been reported at these advisory board meetings*. […] *Now that I’m in this group* [i.e. the advisory board], *I get these updates via the meetings and the reports. So that’s fine.”* (EMA expert 15; project with EMA in an advisory role)

*“They* [i.e. the consortium] *don’t send us regular updates because they take advantage of the annual meeting for updating all the stakeholders and the advisory board.”* (EMA expert 16; project with EMA in an advisory role)

Nearly all of the study participants confirmed that the projects they were engaged in had been affected in some way by the SARS-CoV-2 pandemic that was declared by the World Health Organization in March 2020. In most cases, the pandemic was described as having a negative impact overall, being considered the source of unexpected delays in the realization of project goals. For example, many interviewees felt that the lack of in-person meetings that could be organized during the various SARS-CoV-2 waves had weakened the social cohesion within their consortia and undermined the benefits of multi-stakeholder collaboration. Teleconferences were not seen as an equivalent alternative in this regard.

“*It* [i.e. the SARS-CoV-2 pandemic] *certainly had an impact on the quality of the work that we have done, because* […] *you need to get people around the table, you need a lot of brainstorming. And it doesn’t work on the phone, it doesn’t work if you do TCs* [i.e. teleconferences], *it’s just not the same*.” (Project coordinator 21)

Moreover, some projects did not advance at all throughout the first months of the SARS-CoV-2 pandemic because research activities were temporarily halted, trials could no longer recruit patients, or (post-)doctoral researchers were unable to commence their MSCA-linked traineeships due to travel restrictions. Furthermore, both the EMA experts and the members of the consortia were often occupied with SARS-CoV-2-related work,^4^ leaving little time to spend on the projects.

“*Half of our people cannot make this meeting because they have got to do coronavirus stuff, which probably has to take priority, or they have another conflict.* […] *There’s much more pressure on everyone, not just regulators, to dial into two meetings at a time*.” (EMA expert 1)

“*There were all these research opportunities around COVID-19 which people really felt* [it] *important to engage in, so it was like competing research basically, in many aspects. So you can see that COVID-19* […] *took away also attention from what could be done for our project*.” (Project coordinator 22)

As a result of their projects falling behind schedule, multiple consortia requested or planned to request an extension of their previously agreed upon timelines to IMI or to the European Commission.

“*We had to extend a little bit the deadlines. And this was well respected and well understood by IMI, and it was very, very carefully discussed because we didn’t want to extend it too broadly, because we’d set momentum in the project, and we wanted to make sure we finished up properly with quality deliverables. But it was, I think, a reasonable extension*.” (Project coordinator 18)

However, not all projects were negatively affected by the SARS-CoV-2 crisis. On the contrary, several of the participants stressed that the pandemic had actually demonstrated the scientific relevance of their projects and in some instances even led to strategic and operational plans being adapted to include more SARS-CoV-2-focused undertakings. The need for rapidly generating robust data to inform the treatment of patients infected with SARS-CoV-2 and for monitoring the safety and effectiveness of the various vaccines has underscored the importance of projects like ECRAID-Plan, EHDEN, EU-PEARL, VAC4EU, and WEB-RADR 2.

“*And I must say that COVID-19 by itself, the pandemic, really gave a boost actually to the project*. […] *It really triggered a lot of activities that were not planned initially in the project*.” (Project coordinator 15)

In spite of any SARS-CoV-2-induced uncertainty, nearly all of the interviewees believed that the projects they were or had been involved in were on track to accomplish their objectives within the foreseen (extended) timeframes or had already delivered on them.

*“I think the deliverables which were set were delivered in a way.* […] *You can discuss further about the quality of the output, or, more than the quality, the impact of the output, but the deliverables are there.”* (EMA expert 3)

“*Things are progressing. The architecture of the project is such that the different leaders of the different work packages are very conscious of their time scales.* […] *I think they are very keen on pushing to achieve the time scales originally envisaged*.” (EMA expert 6)

“*It seems that the project is on track, apart from the COVID-related delays.*” (EMA expert 15)

Three EMA experts that were interviewed were more skeptical about the projects they were following, expressing doubt that the ambitions of the consortia or their own aspirations for the projects would be achieved in the end, regardless of any delays incurred as a consequence of the SARS-CoV-2 pandemic. Nevertheless, they also either acknowledged that their interpretation of their project’s anticipated outputs may be premature or recognized that these outputs could still prove to be useful even if they do not meet initial expectations.

*“If it does fail, then you can do a gap analysis and say: ‘the reason it failed is that we haven’t had enough investment in this area or that area.’ And* [if the project fails to meet its objectives,] *that will be one of the outputs we’ll get from it, to know where the gaps in the scientific understanding at the European level are.”* (EMA expert 1)

Overall, the goals set by the projects were perceived as neither too modest nor too ambitious, being viewed as realistic instead.

### 3.3. Views on the relationship and communication between EMA and the project consortia

EMA’s involvement in the projects always originated from a direct request made by the consortia that was often relayed by an individual within the Agency who belonged to the personal network of one of the consortium’s members. Subsequently, every project was subjected to the standard procedure that the Agency has in place to determine whether and how it should become engaged in external research activities (8). Despite its openness to being approached early on, EMA only committed to engaging with consortia after they successfully passed the competitive stages of their grant applications, in accordance with its policy to ensure a level playing field amongst applicants (12).

“*I knew a senior scientist* […] *that has been working at EMA*. […] *And through her, we actually connected with EMA. We went through a very complex process of getting the collaboration approved. It wasn’t easy. I think it took several months, if not more than a year, but then we had the approval from EMA for the project. But everything was channeled through this scientist*. […] *So without this contact, I would have not known how to proceed, honestly*.” (Project coordinator 16)

“*I don’t know what was discussed in the earlier stages.* […] *There’s a limit as to how early, because of the bidding system, we can get involved in these projects*.” (EMA expert 6)

“*We have some contacts* [within EMA]. *And when we contacted them, they said: ‘Okay, it’s interesting. But we don’t want to commit to participating in the consortium. So you can mention that the EMA is interested if the project is funded.’ And then once the project was funded, we came back to the EMA, and this is the regular procedure for the EMA. Then they* [i.e. EMA] *asked us to write a letter to invite them and to explain what is expected from the EMA and eventually they decided to jump in to the project*.” (Project coordinator 10)

The EMA staff members participating in this study generally described their working relationship with the project consortia using positive terms, including “constructive,” “collaborative,” and “smooth.” Vice versa, the coordinators of the projects were overall very pleased with the collaboration as well, praising the professionalism and the expertise exhibited by the Agency’s staff.

“*The relationship was very professional. It was very constructive. It was very open. I have a good impression of the whole relationship with the external stakeholders. It’s very focused, to the point and on the topic*.” (EMA expert 7)

*“It was a very smooth collaboration. It formed the basis of new professional relationships.* […] *So very smooth, very professional, but at the same time, open and friendly. So it didn’t feel as if the Agency was under pressure or that they* [i.e. the consortium partners] *were increasing the complexity of the workload because of difficult communication.”* (EMA expert 14)

*“The relationship is a trusted one, but also an honest one. And if there’s anything that happens that is felt to be really borderline, then that is raised in an open and constructive way. So I think the trust factor was there throughout the project.”* (Project coordinator 18)

“*There is a lot of accuracy and quick responses, so I think that that is working well. So it seems that the people from EMA are working really hard. And it’s also detailed responses and questions, so I appreciate the time they take to review the deliverables and the comments they provide. They show expertise, and it’s nice*.” (Project coordinator 22)

However, several of the coordinators admitted that on occasion, they found it difficult to obtain feedback or to get a timely response to their project-related queries from their EMA contact point.

“*They have been at least invited* […] *to give a reflection on what we present, so a reflection on the progress, but they have not been able to do that so far. I think we also sent around the report, and I also don’t think that we got a written reflection*.” (Project coordinator 1; project with EMA in an advisory role)

“*Of course, I would have wished sometimes that* [name of EMA expert] *would have maybe responded sometimes faster too, but I know how life is*. *I’m also very busy. I think I sent him an e-mail some time ago, and then I didn’t receive a response*.” (Project coordinator 2; project with EMA as a partner of the consortium)

The frequency of communication between the EMA experts and the consortia was strongly project-dependent. As mentioned earlier, for some projects, the Agency only interacted with the consortium a few times per year, during scheduled meetings of the advisory board or the steering committee. Conversely, for other projects, the project partners reached out to EMA much more often, sometimes on a weekly basis. Communication was naturally less sparse when the Agency was part of the consortium, but its intensity varied widely even for projects in which EMA did not contribute directly to any of the work packages. Exchanges between the Agency and the consortium mainly occurred through e-mail or during teleconferences.

“*The* [advisory] *board basically meets once a year, so it’s not very intense. So maybe we have periods before and after that meeting where there’s a lot of exchange, but otherwise it’s quiet during the year*.” (EMA expert 10; project with EMA in an advisory role)

“*At the moment*, [communication happens] *at least weekly. So, the different work packages, and I’m involved in nearly all, have different meetings. So, for instance, this week, I had a meeting yesterday*. […] *There’s another meeting today, which unfortunately I can’t attend because I have clashing meetings. I would say on average weekly*.” (EMA expert 6, project with EMA as a partner of the consortium)

“*On the one hand* [there] *were the teleconferences and meetings with presentations* […]. *So that was one way of communication. The other one was in between those online meetings, where it was just simply e-mail exchanges*.” (EMA expert 7; project with EMA in an advisory role)

In most projects, the degree of interaction between EMA and the consortium fluctuated during the course of the project, reaching a peak whenever regulatory advice or action was needed. Multiple EMA experts remarked that the Agency’s input is especially relevant at the beginning of a project, to steer the consortium in the right direction and to assess whether any EMA support tools could eventually be used. This was also why they stressed that the Agency should ideally be involved as early as possible, a recommendation which the project coordinators seemed to have largely followed.

*“So, we did a lot at the very beginning, when we were writing the proposal, and when the proposal was approved and we had to go through the details of having also EMA recognizing and approving the proposal and the agreement. More recently, we haven’t had any direct interaction, because of course, on our side, we didn’t have any immediate need, and the circumstances were not really requiring an immediate interaction with EMA*.” (Project coordinator 16; project with EMA as a partner of the consortium)

*“To be able to work together efficiently so that they do research which is relevant for us*, […] *I think they have to come to us at the design stage of the projects. So very early on, we have to make them understand: this is relevant to us, this is not relevant to us. And then, if it’s clear up front, then things can move on.”* (EMA expert 17)

### 3.4. Views on EMA’s input into the projects

The input that EMA experts provided into the projects was primarily determined by the Agency’s role in them. For projects in which EMA sat on the advisory board or an equivalent body, the experts’ contributions included providing expert regulatory guidance, reviewing documents drafted by the consortium, attending virtual or physical meetings organized within the context of the project, and liaising between project partners and experts belonging to the EMRN. For projects in which EMA was a formal member of the consortium, the Agency’s delegate also contributed to the realization of the project deliverables and to the dissemination of the research findings through scientific publications and presentations at international conferences. If EMA committed to hosting one or more (post-)doctoral researchers as part of a project, then the staff member who was assigned to the project acted as their mentor, making sure that they had everything they needed to succeed within the Agency.

Although they still perceived the overall workload as manageable, many of the study participants from EMA felt they spent more time on their projects than initially anticipated. Consortia frequently expected or wanted EMA to invest more time and resources into their projects than its role foresaw. However, this was not always possible from a practical point of view.

“*I am putting more time in than we’d originally committed. We’d costed it and I’m going over the time that I had committed according to those costs.*” (EMA expert 6; project with EMA as a partner of the consortium)

“*The amount of work that we can do and that we do is very dependent of the goodwill of the people. We are working more than our* [allotted] *time just for that. All of us are working much more than our* [allotted] *time, because we are interested*.” (EMA expert 4; project with EMA in an advisory role)

*“I received an e-mail last week, saying ‘We* [i.e. the consortium undertaking the project] *want you, we want* [name of one of the interviewee’s colleagues], *and we want* [name of another one of the interviewee’s colleagues].’ […] *That’s all well and good, but all three of us, that’s quite a lot of Agency resources to spend for that kind of thing.”* (EMA expert 1; project with EMA in an advisory role)

Very few of the projects included in this study had utilized or planned to utilize any of the tools that EMA has available to facilitate the development of novel medicines and methodologies, such as scientific advice (13), qualification advice and opinions (14), and ITF briefing meetings (15). Many of the project coordinators were unaware of the existence of the Agency’s support tools or lacked knowledge about the conditions under which they could be employed. Upon learning more about them, several of the interviewees in question indicated that they would look into them to see if they could be applied in their projects. Projects in this study that explicitly intended to use or that had already used such regulatory mechanisms at the time of the interviews were ConcePTION (ITF meeting, qualification advice and opinion), EU-PEARL (ITF meeting) and PREFER (qualification advice and opinion). The scope of some projects did not allow them to exploit all of these mechanisms, their coordinators believed.

“*I don’t know actually about this Innovation Task Force. So I’m not aware of these tools*.” (Project coordinator 8)

“*I haven’t yet explored this. I did not know that these tools were available*. […] *I will definitely go more into the details of this*.” (Project coordinator 16)

“*When you mention scientific advice, what do you mean? Is it just an informal interaction?*” (Project coordinator 23)

All of the project coordinators who were interviewed felt that EMA’s involvement in their projects had increased or would increase the scientific relevance of the project outputs. Being able to leverage the Agency’s insights, experience, authority and visibility were stated as major benefits of having EMA on board as an advisor or a contributor to a project. Multiple coordinators even went so far as to describe the Agency’s input into their project as essential.

“*I think they* [i.e. EMA] *opened doors for us*. […] *So, thanks to those interactions, for example, we were involved in the dialogue with* […] *the National Competent Authorities*.” (Project coordinator 3)

“*The fact that EMA was on board sent out a very important message at European level but also globally.* […] *The authority, the respect, the expertise that the Agency stands for bring that certain sort of calibre into the project, which was very good as well.* […] *So the added value* [of EMA’s involvement] *was both political and linked to the subject matter per se.*” (Project coordinator 18)

“*At the end, the evidence that we generate needs to be acceptable by EMA.* […] *So it’s really important that there is buy-in*. […] *It’s better to have them on board while we create the solutions, rather than creating the solutions and see then whether they are acceptable* [for EMA].” (Project coordinator 22)

The EMA experts expanded on this by emphasizing that academics are often not very familiar with the regulatory principles and procedures by which the medicinal products and the methods they develop are scientifically evaluated and approved by regulators. By being present at the table and offering regulatory guidance, EMA can help the consortium members understand what they need to do to maximize the chances of their research outcomes being translated into innovations which are accepted and used by others in their field and which can reach patients.

“*From our recent interactions in the different projects, I realized that researchers have very little idea of what we’re doing on a daily basis and where our interest lies*. […] *So I think it’s just to have the opportunity for us, the regulators, to explain to researchers what we do and where our interest lies*. […] *Given the binary nature of the decisions we have to make that have a strong impact on public health, we look at things differently. And I think to explain that to researchers, that’s important*.” (EMA expert 17)

“*At the beginning, I had the feeling they were doing something which was really not aligned with what is really needed*. […] *And bringing this a bit towards more tangible, regulatory-valued outcomes, I think this was very useful for them. And I hope that* [going forward,] *we state, ‘okay, if this needs to be, or is relevant, or should be relevant for patient access to medicines or to treatments, then this needs to follow certain regulations.’ And in this respect, I think it’s good that we participate*.” (EMA expert 5)

“*There was a lot of input that we brought in, to developers, to academic groups doing this type of research, about the regulatory thinking*. […] *And also then in terms of the outcome, that we understand where this is coming from. And that will help uptake in regulatory decisions*.” (EMA expert 16)

### 3.5. Views on the outputs from the projects

The projects had produced or aimed to produce a diverse set of scientific deliverables that are or will be relevant to the development, evaluation or use of medicines or diagnostics, including (but not limited to) new or improved (a) medicinal products (e.g., AVANT), (b) monitoring and reporting tools (e.g., WEB-RADR 2), (c) methodological standards and frameworks (e.g., PARADIGM, PERMIT, and PREFER), (d) research infrastructures and networks (e.g., c4c, ECRAID-Plan, and VAC4EU), and (e) educational concepts, materials and programs (e.g., MINDED, PEARRL, and STARS). The project coordinators believed that these deliverables had or would have major implications for the field of regulatory science. More specifically, they thought that the outputs of their projects would directly or indirectly stimulate and accelerate the development of innovative treatments, vaccines, and diagnostics by facilitating the generation of evidence that satisfies the strict assessment criteria employed by regulators and by filling the gaps in the current regulatory framework. The value of the deliverables was also explicitly recognized by the EMA interviewees.

*“I hope that we really get out with some pilots, some best practices*, […] *to really establish all those best practices or pilots* […] *as instruments, supporting regulation, and supporting development of new and especially innovative pharmaceutical medicinal products and devices.”* (Project coordinator 14)

*“The project has identified* […] *some regulatory gaps.* […] *And this will be the contribution to regulatory science.* […] *At the beginning, we said that the project is about establishing methodological standards* […]. *And now, I add, it’s about establishing methodological and regulatory standards. Because, I think*, […] *you cannot speak about methodology in Europe if you don’t consider also the regulatory framework.”* (Project coordinator 10)

*“I think from the output, it was really helpful to us. As I said, the results are really important in the context also of our operational activities*. […] *The output is really feeding into our day-to-day activities or has given us important information on how to proceed in certain areas*.” (EMA expert 9)

With respect to the dissemination of the outputs, most projects featured work packages that were dedicated to devising communication strategies which would widen the reach of the deliverables as much as possible. As part of such strategies, the results of nearly every project had been or were going to be published in open-access journals and promoted via the projects’ websites and social media pages as well as through presentations at international conferences, workshops and symposia.

*“We do have a concrete dissemination plan, and the next steps will be the workshop* [.]. *Then we will have a conference*, […] *and also some further publications*.” (Project coordinator 11)

Nevertheless, some EMA experts remarked that scientific publications alone were not sufficient to guarantee the sustainability of the project outcomes, again underlining the importance of using regulatory mechanisms such as the EMA qualification procedure to increase the awareness and the impact of the research findings and to ensure their longevity.

“*The risks are always that these things become nice academic papers, and that once the money runs out, the PhDs are written up and everybody goes home. And that this has been a nice endeavour, but it’s going to stay on paper*. […] *So, is this* […] *going to create a culture change?*” (EMA expert 2)

One of the EMA experts also proposed that the consortia would present their projects to the Agency’s guideline drafting groups upon completion so that regulators would be able to assess for themselves whether the outputs could inform or support their decision-making.

*“What I said earlier* […] *is for these researchers* [i.e. the academics undertaking a project] *to be part of guideline drafting groups*. […] *When I say guidelines, I think any regulatory recommendations*. […] *We could have someone who has done some research in the area and keeps us on track and says, ‘Yeah, but are you aware of this and that?’* […] *It could be that as part of recommendations*, […] *we have three presentations from different projects that have happened over the last three, five years*.” (EMA expert 17)

### 3.6. Reflections on the projects and suggestions for improving EMA engagement

When the EMA staff members participating in this study were asked to comment on how they personally perceived the balance between the input they provided into a project and the outputs for the Agency (potential or actual, depending on the project’s status), there was near-universal agreement that the latter outweighed the former, meaning that the knowledge and the deliverables generated were considered more valuable than the time and resources invested, from the EMA experts’ perspective. A side-by-side overview of the Agency’s contributions to and gains from the projects according to these interviewees is presented in Table 2.

**TABLE 2:** High-level overview of EMA’s contributions to and gains from the projects, according to the Agency experts participating in this study.

EMA contributions	EMA gains
Guidance on use of EMA support tools	Awareness of innovative research and of its hurdles and opportunities
Regulatory knowledge and guidelines	Learnings from dialogue with innovators
Regulatory data	Identification of gaps in regulatory science
Experience from previous involvement in other regulatory science projects	New or improved methodologies or standards
Active contribution to work packages	Visibility through dissemination of results (e.g., publications and conference presentations)
Insights into regulatory thinking and activities	Research network and access to expertise
Recommendations derived from consultations with other regulators	Outputs addressing regulators’ priorities and patients’ needs

Note that the extent of EMA’s contributions to a project is dependent on its role in that project, so not every type of contribution listed here will be provided to all projects.

*“It* [i.e. the overall balance between input and output] *is positive.* […] *The input from our side is light. But the possible implication for what we do is quite substantial. So, to be able to be informed and to have our say is extremely important.”* (EMA expert 8; project with EMA in an advisory role)

*“We are on the winning side, because we share our experience, we also get things back, and really disseminate in the broader sense. And that’s really in our core business, our aim is really to* […] *engage with academia, so that’s totally in line with our responsibility. I don’t think it’s a loss.”* (EMA expert 11; project with EMA as a partner of the consortium)

Similarly, the project coordinators were all convinced that their projects had or would have a high “return on investment” overall.

*“I think the outputs are significantly more valuable than the time and effort that we put in.* […] *The initial investment was a relatively small one compared to the benefit that we’re getting out.”* (Project coordinator 19)

*“On balance, the input and output were just about what I expected, and in that case it was a success. We achieved what we needed to do, everybody had a positive experience, we committed to a certain amount* [of output] *and we delivered on that with minor deviations that we were able to mitigate and resolve.”* (Project coordinator 20)

However, several of the EMA experts underscored that although they saw the balance as positive, this did not imply that there was no room for further improvement: according to them, additional efforts could still be undertaken by some of the consortia to disseminate the project outcomes more broadly and to safeguard their sustainability.

“*I think it’s very important what the project has achieved. We are very pleased with it. The input from EMA has been good, well-justified in that sense, but we can improve things, to optimize our return*.” (EMA expert 2; project with EMA in an advisory role)

*“I think the effort that the Agency put in that project could have been more efficient*. […] *Better information sharing, that would make the overall balance more positive*. […] *The benefits were more localized, were more specific to a very narrow group of people who are perhaps directly involved, but did not spread out to the extent that they could have been*. *I think overall it was a positive experience*. […] *But things can always be done better*.” (EMA expert 14; project with EMA as a partner of the consortium)

**FIGURE 2:**
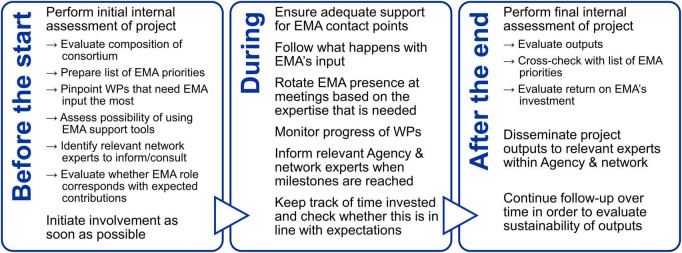
Overview of the actions proposed by some of the Agency experts interviewed that EMA is already taking but can take more stringently and systematically across a project’s life cycle to increase the regulatory relevance of the project outputs. WP, work package.

Based on their experience, the EMA experts interviewed suggested various actions that could be taken to improve the relevance of the project deliverables to regulators (Figure 2), including the creation of a list at the start of the project that would serve to make the project partners aware of where the Agency’s priorities lie with respect to the field of regulatory science. This list would eventually be revisited to evaluate whether any of these priorities were met. Another suggestion that was made was to actively follow what happens with EMA’s guidance after receipt by the consortium. This would also enable the Agency to prioritize its interactions with consortia that are responsive to its feedback. Furthermore, it was proposed that EMA would perform an assessment of the academic and wider uptake of these outputs a few years after the end of the project in order to evaluate their sustainability.

*“We could have our own agenda for each project, so that we then maximize our engagement, because we know what we want that project to deliver for us, being a bit selfish*. *Because you could do that if you would be* [present] *in the planning period* [of the project]. […] *But you are not there then, because we have to be independent. So when you join, everything* [with regard to the planning of the project] *is done, but still, you could really then identify* […] *the areas where we need to be there*, […] *for those predefined objectives.”* (EMA expert 10)

*“To probably clarify better what is EMA’s involvement in terms of time but also in terms of leverage. So is what we are saying or advising really taken into account or not?* […] *I think it would help, because it would also help to prioritize our time.”* (EMA expert 8)

*“What I see as important is that we follow up on the project implementation.* […] *You close the projects, you have a proposal for sustainability to ensure that we have a plan to implement* [the outputs], *but I would really like to see later on a follow-up of this implementation, even a phase two project, to do the assessment on how it was really implemented.* […] *Do a real proper analysis on defined criteria. I think this would really show the benefits and would perhaps also show then where the weaknesses are.”* (EMA expert 9)

Several of the interviewed EMA experts requested additional internal support, usually in the form of administrative assistance or organizational help. For example, some participants expressed that they found it difficult to keep track of when project meetings were being held which required their attendance or to identify persons within the Agency’s network who could give relevant input on specific issues being discussed during these meetings. Moreover, in some projects where EMA was a partner of the consortium, the Agency’s legal office was involved to resolve issues relating to requests for signing non-disclosure agreements, which EMA does not do, or for modifying the standard grant agreement.

*“Definitely all the administrative aspects of the collaboration. You might need* [i.e. be asked by the consortium] *to sign contracts or confidentiality agreements. The practicalities about* […] *how we meet, how we organize a teleconference, for example. You will need some secretarial support.”* (EMA expert 14)

*“In areas where I don’t have a strong scientific background*, […] *being able to drop an e-mail to someone unconnected to a project, a link that the Agency has, and say, ‘Can you point me in the direction of three or four papers?’* […] *It’s something that I’ve always had in my mind* […], *that when we are trying to identify relevant expertise within Europe, we need to work with the* [Agency’s] *Academic Liaison Office.”* (EMA expert 1)

## 4. Discussion

In this study, we evaluated perceptions of the added value of EMA’s participation in 21 externally funded regulatory science projects among the coordinators of these projects as well as among the Agency’s staff members who were involved in them. Through interviews with these stakeholders, we found that EMA’s involvement in the projects benefited not only the consortia that undertook them, but also the Agency itself by supporting its strategic activities. The deliverables generated were deemed highly relevant from the regulatory perspective of the EMA experts interviewed. Based on suggestions from the interviewees, several recommendations were formulated to improve EMA’s future engagement in external research projects and to increase the chances of their outputs being translated into innovations that can improve public and animal health. The results of this study will contribute to the Agency’s assessment of its process for participating in such projects and to the realization of the first deliverable in EMA’s Academia Collaboration Matrix action plan (15).

Regardless of whether they had already been produced or were still forthcoming, the deliverables of the projects we examined were expected to have a substantial impact on EMA procedures and guidelines, thereby informing developers and thus ultimately facilitating the generation of regulatory-grade evidence that would underpin the development of novel treatments, vaccines, or diagnostics. Nevertheless, it will still take some time before the full extent of this impact becomes clear. Once outputs have matured and their value has been demonstrated, the Agency can assess whether and how it should implement them into the regulatory framework. This potential implementation provides opportunities for projects to ensure the sustainability of their deliverables. For example, the results of the PARENT Joint Action, which ran from 2012 to 2015 with EMA participation and aimed to provide EU Member States with tools to support the setup of interoperable patient registries (22, 23), informed the conception of the Agency’s guideline on registry-based studies that was issued in 2021 (24). Overall, our findings complement those of an IMI-commissioned study which investigated the wider socio-economic implications of IMI1 project outcomes (16).

The achievements realized under the auspices of IMI prompted the European Union to establish a new public–private partnership with an expanded scope, namely, the Innovative Health Initiative (IHI) (25, 26). IHI is the successor to IMI yet does not exclusively focus on research in the domain of pharmaceutical sciences, having adopted a broader, health-oriented agenda instead. Its inclusion of additional commercial partners from the medical technology, biotechnology, and digital technology industries allows for the financing of cross-disciplinary projects which tackle a wider spectrum of healthcare-related topics and which would not have been eligible for IMI funding. EMA is represented in IHI’s Science and Innovation Panel (27), an advisory body giving input on IHI’s funding calls, work plans and strategic objectives. The Agency will continue to consider requests from consortia to get involved in IHI projects that are compatible with its mission (1) to foster scientific excellence in the evaluation and supervision of medicines for the benefit of public and animal health in the EU, based on the evolving practices and the established process for such engagement (8). Likewise, EMA remains interested and available to contribute to research activities (e.g., MSCAs) taking place within the context of Horizon Europe (28), the key research and innovation program which succeeded H2020.

EMA recently published a list of more than one hundred research topics in the field of regulatory science which are of particular importance to the Agency’s work. The so-called Regulatory Science Research Needs initiative (29) outlines specific areas for which regulators are currently faced with knowledge gaps that complicate their decision-making. These needs were identified through interviews and consultations with the chairs of EMA’s scientific committees and working parties, as well as with external experts and representatives of key stakeholder groups. The Regulatory Science Research Needs initiative is mainly targeted toward researchers and funding organizations, enabling them to conduct or support research that addresses questions for which the Agency is seeking answers. Consortia looking to set up their own projects are encouraged to browse through the list of needs and check whether there are any topics mentioned that they would like to tackle as part of their planned tasks. If this is the case, it will be taken into consideration by EMA when deciding to get involved in the project, and the coordinating team can contact the Agency’s Academia Liaison Office for further discussion.

Few of the projects covered in this interview study had used or intended to use any of the support tools that EMA has available to aid the development of novel medicines and methodologies, including scientific advice, qualification advice and opinions, and ITF briefings. While these services offered by EMA may not be applicable to every project, our results point toward a lack of awareness among academia-affiliated researchers^5^ of how such regulatory mechanisms and pathways can be properly exploited, a finding which was confirmed in a series of surveys (30) carried out by the STARS consortium. This indicates that regulators need to improve the way they communicate about mechanisms of this kind. In fact, it is part of the motivation of EMA when engaging in external projects to inform the consortia about its support tools and to motivate them to make use of these opportunities for interaction, as they enable discussion of a specific product or technology, for which EMA formally convenes multiple topic experts from across the EMRN to learn, share experience and provide guidance that applies across the EU. Some of the project coordinators believed that EMA’s involvement in their projects gave them access to the EMRN, but this is a common misunderstanding and contrary to the Agency’s systematic communication that the participation of an EMA expert does not represent engagement with the EMRN, which requires the use of one of the Agency’s support tools. A document prepared by IMI helps clarify how researchers can take optimal advantage of these tools (31). For the deliverables of IMI projects to have an impact on the regulatory assessment of novel methodologies, a positive qualification opinion issued by the CHMP is often considered valuable by regulators. An opinion of this nature establishes the acceptability from a regulatory perspective of a new method for application in a research and development context, whether in clinical or in non-clinical studies (15, 32). This method can then be used to develop medicines that can benefit patients. Over the past decade, a considerable number of IMI project consortia have sought early interaction with regulators to ensure that the research they planned to undertake would lead to such impactful outcomes (33).

In 2020, IMI2’s Scientific Committee formulated several recommendations for involving regulators in projects initiated within the context of a public–private partnership (34). Researchers are encouraged to follow these recommendations when setting up a project in which they would like EMA to participate. For example, it is advised that consortia develop a dedicated strategy for interacting with medicines regulators and outline it in their research proposals. It should be stressed here that as part of their involvement in a particular project, to avoid conflicts of interest, EMA experts will not sign any non-disclosure agreements. When EMA sits on the advisory board, the staff member in question should be able to provide the regulatory input required without seeing data that are commercially confidential. To mitigate the theoretical risk of project results leaking and hence their publication being compromised, EMA relies on the confidentiality provisions in the EU Staff Regulations (35) as well as in the Agency’s Code of Good Administrative Behavior (36) and Code of Conduct (37).

This study has limitations, which should serve as caveats to anyone using our results. Firstly, the projects that were included were mostly still ongoing at the time of the interviews and were all relatively young, with the majority having been launched in 2019 or later. This indicates that our interviews could not reflect the full experience of the participating coordinators and EMA staff members. In addition, our sample covered fewer than half of all externally funded regulatory science projects that EMA has engaged in thus far. We chose to focus on a subset of recent projects for practical reasons, in particular to facilitate the process of recruiting the interviewees. Ideally, our study should be repeated and expanded upon in the future, when there is more clarity on the academic and wider uptake of the project deliverables. This follow-up research could then explore the overall impact of regulatory science projects on medicines development and regulation. Secondly, the methodology we employed was qualitative in nature, which means that we made no attempt to quantify any of the information that the participants shared with us. A more comprehensive investigation would have also examined quantitative metrics (e.g., the number of publications following from the projects, the number of hours the EMA experts spent working on the projects). Thirdly, as this was a study that was initiated and supervised by EMA, the project coordinators may have felt inhibited to express their true opinions about the Agency’s involvement in their projects, for fear of experiencing negative repercussions. However, this effect was at least partially counteracted by having an external researcher who was not an EMA staff member conduct the interviews and by assuring the interviewees that the original, non-anonymized transcripts would not be shared with anyone from the Agency that did not belong to the research team. Fourthly, since only one person (RS) was responsible for carrying out the interviews and analyzing the data, our findings may have been affected by researcher bias. Lastly, many of the projects that were featured in the present study were large and complex, and the consortia that were undertaking them often had a decentralized organizational structure. Consequently, the project coordinators were not always aware of the status of each individual work package and could therefore not give detailed answers to some of the questions. A follow-up study could address this shortcoming by interviewing additional consortium partners, including industry representatives for IMI projects, which accounted for most of the undertakings making up our sample.

## 5. Conclusion

In this interview study, we found that EMA’s involvement in recent research projects that tackled regulatory science issues and were coordinated by external stakeholders such as academic researchers not only benefited the consortia undertaking those projects, but also the Agency by informing its strategic activities. From the perspective of the project coordinators, being able to leverage regulators’ experience and guidance were the most significant benefits of having an EMA expert on board as an advisor or a contributor to their projects. From the point of view of the Agency’s staff members involved, the deliverables generated by the projects, which comprised new or improved medicinal products, monitoring tools, methodological standards, research infrastructures, and educational materials, were of high regulatory value. Nevertheless, few projects made use of the services offered by EMA to support the development of novel medicines and methodologies. To make its participation more worthwhile, the Agency could continue assessing how and to what extent it engages with consortia, strengthen its decision-making in this respect, and monitor ongoing projects more actively to leverage emerging outputs. Funders and regulators could launch coordinated efforts with the aim of stimulating consortia to seek formal regulatory engagement and to exploit the tools and procedures that are available in this regard, which should render their projects’ results more impactful. Collaboration between regulatory authorities and academia is of paramount importance for the successful translation of academic research outputs into scientific innovations that can be used by regulators to improve public and animal health.

## Data availability statement

The raw data supporting the conclusions of this article (i.e., the anonymized interview transcripts) will be made available by the authors, without undue reservation.

## Ethics statement

The studies involving human participants were reviewed and approved by the Ethics Committee Research UZ/KU Leuven. The patients/participants provided their written informed consent to participate in this study.

## Author contributions

CV and RH designed and implemented the strategy for EMA’s interaction with externally funded regulatory science projects. RS, LL, JL, and IH contributed to the conception and design of the study. RS conducted the interviews, analyzed and interpreted the interview data, and drafted the study manuscript. RS, MM, and BC transcribed the interviews. RS, MM, BC, LL, CV, RH, and IH revised the draft manuscript and approved the submitted version.

## Footnotes

1 EMA defines regulatory science as the range of scientific disciplines that are applied to the quality, safety, and efficacy assessment of medicinal products and that inform regulatory decision-making throughout the lifecycle of a medicine, encompassing basic and applied biomedical and social sciences and contributing to the development of regulatory standards and tools (76).

2 Such conflicts may for example arise when a consortium goes on to make use of one of EMA’s regulatory support tools or procedures.

3 Note that some projects were jointly coordinated by commercial (e.g., pharmaceutical companies) and non-commercial (e.g., universities) parties. For these projects, only the latter were targeted for recruitment, because the study mainly aimed to capture the perspectives of academic stakeholders with whom EMA has engaged.

4 During the SARS-CoV-2 pandemic, EMA operates under the principles set out in a dedicated business continuity plan (77).

5 Note that the familiarity of commercial participants of public–private research projects with EMA’s support tools was not addressed in this work.
