# Supplementary material for: Involvement of the European Medicines Agency in multi-stakeholder regulatory science research projects: experiences of staff members and project coordinators
Source: Front Med (Lausanne). 2023 May 30;10:1181702. doi: 10.3389/fmed.2023.1181702 (PMC10267977; doi:10.3389/fmed.2023.1181702)
Supplement: Supplementary file 1 [file Table_1.DOCX]

| **Step** | **Description** |
| --- | --- |
| Transcribing the interviews | After each interview has been concluded, it is transcribed verbatim, meaning that everything that was said or expressed during the interview is written down word for word, including filler words, mispronunciations, laughter, sighs, repetitions of words, etc. Adequate spacing is left between the individual lines of each transcript to create room for making notes later. |
| Getting familiarized with the content of the interviews | Familiarization with the interviews is mainly achieved through reading the transcripts. When something is still not clear after reviewing the transcripts, the researcher responsible for analyzing the data (ideally the person who conducted the interviews) will listen to the audio recordings again. During this familiarization step, the spacing between the transcript lines is used to write down thoughts, impressions and remarks (e.g. an observed contrast between the answers of different interviewees). |
| Coding the transcripts of the interviews | The transcripts are read line by line by the researcher undertaking the data analysis. Concurrently, a label (a so-called ‘code’) is added to text fragments to summarize the information contained within them. The aim of coding is to classify all data collected during the interview so that they can be compared systematically with data obtained from other interviews. If a study is primarily deductive in nature, the coding list is predefined to a certain extent, since the codes are then based on the specific topics addressed in the study. In the context of structured interviews, that means the codes largely overlap with the question topics. Additionally, open coding can be applied for the more inductive aspects of a study (e.g. participants’ experiences, views, concerns). |
| Developing a working analytical framework | In this step, the coding system gradually improves as the first transcripts are analyzed. Related codes are grouped together into categories using a computer-assisted qualitative data analysis software (CAQDAS) tool, i.e. a computer program specifically designed for qualitative data analysis. This approach produces a so-called working analytical framework. |
| Applying the working analytical framework | Once a working analytical framework has been established, it is applied to all subsequent transcripts by linking the appropriate codes to specific sections of text through the use of a CAQDAS tool. The framework itself is still subject to change, meaning that additional codes and categories can be created if necessary. |
| Charting of the data into the framework matrix | The data contained within each transcript are summarized and mapped in an Excel^®^ spreadsheet, where the columns represent cases (i.e. individual interviewees) and the rows display the different codes that were used within the analytical framework. Each cell then contains the relevant text fragments that were categorized under these codes. The resulting table is known as the framework matrix. |
| Interpreting the data | The final framework matrix is examined in detail to identify connections and patterns between the answers provided by different interviewees. Upon completion of this step, the study findings can be documented, and conclusions can be made with respect to the research questions. |

Supplementary table 1: Overview of the framework method for analyzing textual data derived from interviews (1,2).

References

1. Gale NK, Heath G, Cameron E, Rashid S, Redwood S. Using the framework method for the analysis of qualitative data in multi-disciplinary health research. BMC Med Res Methodol (2013) **13**:117. doi: 10.1186/1471-2288-13-117

2. Spencer L, Ritchie J, O’Connor W, Morrell G, Ormston R. “Analysis in Practice” In: Ritchie J, Lewis J, McNaughton Nicholls C, Ormston R, editors. Qualitative research practice: a guide for social science students and researchers. London, UK: SAGE Publications Ltd. (2014). p. 295–343.
