# Supplementary material for: Involvement of the European Medicines Agency in multi-stakeholder regulatory science research projects: experiences of staff members and project coordinators
Source: Front Med (Lausanne). 2023 May 30;10:1181702. doi: 10.3389/fmed.2023.1181702 (PMC10267977; doi:10.3389/fmed.2023.1181702)
Supplement: Supplementary file 2 [file Data_Sheet_1.DOCX]

***Interview guide project coordinators (ongoing projects)***

**Introduction**

- **Present yourself**

*First, allow me to present myself. My name is Robbe Saesen and I am a PhD researcher at KU Leuven working at the Clinical Pharmacology and Pharmacotherapy research unit under the supervision of Professor Isabelle Huys. Within the context of the Collaborating Expert Programme, I am also working at the Task Force Regulatory Science & Innovation of the European Medicines Agency under the mentorship of Jordi Llinares Garcia.*

- **Thank** the interviewee for his/her participation in advance

*I would like to thank you already for taking the time to participate in this interview. Your views, opinions and experiences are very important to our research.*

- Explain **shortly the purpose** of the interview:
  - *With this interview we want to explore your experiences with participating in externally funded regulatory science projects to which EMA has contributed* (keep it brief)
  - [If the interviewee is involved in **one** project] *The interview will take about* ***1 hour.***

[If the interviewee is involved in **two** projects] *The* *interview will take about* ***1 hour and 15 minutes.***

[If the interviewee is involved in **three** projects] *The interview will take about* ***1 hour and 30 minutes****.*

- - *Results will be* *implemented in* ***scientific publications.***
- Put the interviewee **at ease**:
  - *I want to emphasize that there are no right or wrong answers, and that it is no problem if you might not know the answer to a particular question. In that case, it would be great if you could recommend us the name of a contact person who could clarify this specific aspect.*
  - *This interview will be* ***digitally recorded****. This makes it easier for us to process all of the information that you provide us with during the interview. Anything you say today will be processed* ***pseudonymously****, meaning we will not use your name or any identifying information in publications following from this study. The data collected will be stored securely and viewed in unredacted form only by the researchers involved in this project.*
  - *Participation is* ***completely voluntary****. You can* ***withdraw*** *at any point, without having to give an explanation. You do not have to answer any questions during the interview if you do not feel comfortable answering them.*
- *Do you have any* ***questions*** *before we start the interview?*
- *We will start with some* ***warm-up*** *questions. Subsequently, we will turn on the* ***recorder*** *and focus more on the* ***research questions*** *which are organized across five main themes. The first theme concerns the project’s status and planning, the second theme will focus on the partnership with EMA, the third theme will deal with EMA’s input in the project, the fourth theme will address the learnings from the project and the fifth theme will cover some reflection questions.*

**Questions**

I. Introductory questions

1. Could you please start with shortly introducing yourself?
2. How long have you been working in your current position?
3. What are your expectations of this interview?

- *Let’s start with the research questions now. Do I have your permission to turn on the recorder?*

II. Questions about the project’s status and planning

1. What is the current status of the project?
2. What has been the impact of COVID-19 on the project?
3. To what extent is the project on track to realize its deliverables within the foreseen timeframe?

III. Questions about the partnership with EMA

1. When and how did EMA get involved in this project?
2. How would you describe your relationship with EMA within the context of this project?
3. How and how often do you communicate with EMA?
4. How has the scope of your interaction with EMA in this project changed since the start of the project?
5. In your view, what is working well in this project in general and in the partnership with EMA in particular?
6. In your view, what is not working well in this project in general and in the partnership with EMA in particular?

IV. Questions about EMA’s input in the project

1. What specific activities has EMA been involved in for this project? (e.g. agenda setting, document review, document writing, teleconferences, stakeholder liaison,...)
2. Which regulatory tools that the Agency has available to support external stakeholders in their activities have been used in this project so far? (e.g. scientific advice, Innovation Task Force, qualification advice/opinion, etc.)
3. How would you assess EMA’s contributions to the project so far in terms of their added value?
4. To what extent do you think EMA participation in this project is still relevant at this point in time?
5. [If you think EMA participation is still relevant] What exactly can EMA still add according to you?

[If you think EMA participation is no longer relevant] What exactly has changed that makes EMA participation no longer relevant according to you?

V. Questions about the learnings from the project

1. In your view, what are the most important practical learnings for you from this project so far?
2. In your view, what are the most important scientific learnings from this project so far?
3. How have these scientific learnings been shared so far (e.g. publications, workshops, conferences)?
4. What do you think are the implications of these learnings for the field of regulatory science?

VI. Reflection questions

1. In your opinion, how do the time and resources invested into this project weigh against its outputs?
2. Looking back with the knowledge that you have now about this project, what, if anything, would you have done differently?
3. How has your involvement in this project shaped the way in which you will approach similar projects in the future?
4. To what extent have you interacted with EMA’s Academia Liaison Office for this project?

VII. Round-up questions

1. Do you want to **add** anything else to what you’ve already said?
2. Do you want to **emphasize** something?
3. Do you think we **forgot or overlooked** some relevant questions?
4. Do you have any **questions for me**?
5. Are there any other **persons** involved in this project from your side that we could interview?

- *These were all the questions I had for you. I will turn off the recorder now.*

**Conclusion**

- ***Thank you*** *for your participation.*
- *If you have any other questions or comments, or if you want to get in touch with me, please do not hesitate to* ***contact me.***

***Interview guide project coordinators (finalized projects)***

**Introduction**

- **Present yourself**

*First, allow me to present myself. My name is Robbe Saesen and I am a PhD researcher at KU Leuven working at the Clinical Pharmacology and Pharmacotherapy research unit under the supervision of Professor Isabelle Huys. Within the context of the Collaborating Expert Programme, I am also working at the Task Force Regulatory Science & Innovation of the European Medicines Agency under the mentorship of Jordi Llinares Garcia.*

- **Thank** the interviewee for his/her participation in advance

*I would like to thank you already for taking the time to participate in this interview. Your views, opinions and experiences are very important to our research.*

- Explain **shortly the purpose** of the interview:
  - *With this interview we want to explore your experiences with participating in externally funded regulatory science projects to which EMA has contributed* (keep it brief)
  - [If the interviewee is involved in **one** project] *The interview will take about* ***1 hour.***

[If the interviewee is involved in **two** projects] *The* *interview will take about* ***1 hour and 15 minutes.***

[If the interviewee is involved in **three** projects] *The interview will take about* ***1 hour and 30 minutes****.*

- - *Results will be* *implemented in* ***scientific publications.***
- Put the interviewee **at ease**:
  - *I want to emphasize that there are no right or wrong answers, and that it is no problem if you might not know the answer to a particular question. In that case, it would be great if you could recommend us the name of a contact person who could clarify this specific aspect.*
  - *This interview will be* ***digitally recorded****. This makes it easier for us to process all of the information that you provide us with during the interview. Anything you say today will be processed* ***pseudonymously****, meaning we will not use your name or any identifying information in publications following from this study. The data collected will be stored securely and viewed in unredacted form only by the researchers involved in this project.*
  - *Participation is* ***completely voluntary****. You can* ***withdraw*** *at any point, without having to give an explanation. You do not have to answer any questions during the interview if you do not feel comfortable answering them.*
- *Do you have any* ***questions*** *before we start the interview?*
- *We will start with some* ***warm-up*** *questions. Subsequently, we will turn on the* ***recorder*** *and focus more on the* ***research questions*** *which are organized across five main themes. The first theme concerns the project’s status and planning, the second theme will focus on the partnership with EMA, the third theme will deal with EMA’s input in the project, the fourth theme will address the learnings from the project and the fifth theme will cover some reflection questions.*

**Questions**

I. Introductory questions

1. Could you please start with shortly introducing yourself?
2. How long have you been working in your current position?
3. What are your expectations of this interview?

- *Let’s start with the research questions now. Do I have your permission to turn on the recorder?*

II. Questions about the project’s status and planning

1. What is the current status of the project?
2. What was the impact of COVID-19 on the project?
3. To what extent was the project able to realize its deliverables within the foreseen timeframe?

III. Questions about the partnership with EMA

1. When and how did EMA get involved in this project?
2. How would you describe your relationship with EMA within the context of this project?
3. How and how often did you communicate with EMA?
4. How did the scope of your interaction with EMA in this project change over the course of the project?
5. In your view, what worked well in this project in general and in the partnership with EMA in particular?
6. In your view, what did not work well in this project in general and in the partnership with EMA in particular?

IV. Questions about EMA’s input in the project

1. What specific activities was EMA involved in for this project? (e.g. agenda setting, document review, document writing, teleconferences, stakeholder liaison, ...)
2. Which regulatory tools that the Agency has available to support external stakeholders in their activities were used in this project? (e.g. scientific advice, Innovation Task Force, qualification advice/opinion, etc.)
3. Looking back now, to what extent do you think EMA participation in this project was relevant?
4. [If you think EMA participation was relevant] What exactly did EMA participation add to the project?

[If you think EMA participation was not relevant] What exactly changed from the initial proposal that in the end made EMA participation less relevant than expected?

V. Questions about the learnings from the project

1. In your view, what were the most important practical learnings for you from this project?
2. In your view, what were the most important scientific learnings from this project?
3. How were these scientific learnings shared (e.g. publications, workshops, conferences)?
4. What do you think are the implications of these learnings for the field of regulatory science?

VI. Reflection questions

1. In your opinion, how did the time and resources invested into this project weigh against its outputs?
2. Looking back with the knowledge that you have now about this project, what, if anything, would you have done differently?
3. How has your involvement in this project shaped the way in which you will approach similar projects in the future?
4. To what extent did you interact with EMA’s Academia Liaison Office for this project?

VII. Round-up questions

1. Do you want to **add** anything else to what you’ve already said?
2. Do you want to **emphasize** something?
3. Do you think we **forgot or overlooked** some relevant questions?
4. Do you have any **questions for me**?
5. Were there any other **persons** involved in this project from your side that we could interview?

- *These were all the questions I had for you. I will turn off the recorder now.*

**Conclusion**

- ***Thank you*** *for your participation.*
- *If you have any other questions or comments, or if you want to get in touch with me, please do not hesitate to* ***contact me.***

***Interview guide EMA experts (ongoing projects)***

**Introduction**

- **Present yourself**

*First, allow me to present myself. My name is Robbe Saesen and I am a PhD researcher at KU Leuven working at the Clinical Pharmacology and Pharmacotherapy research unit under the supervision of Professor Isabelle Huys. Within the context of the Collaborating Expert Programme, I am also working at the Task Force Regulatory Science & Innovation of the European Medicines Agency under the mentorship of Jordi Llinares Garcia.*

- **Thank** the interviewee for his/her participation in advance

*I would like to thank you already for taking the time to participate in this interview. Your views, opinions and experiences are very important to our research.*

- Explain **shortly the purpose** of the interview:
  - *With this interview we want to explore your experiences with participating in externally funded regulatory science projects to which EMA has contributed* (keep it brief)
  - [If the interviewee is involved in **one** project] *The interview will take about* ***1 hour.***

[If the interviewee is involved in **two** projects] *The* *interview will take about* ***1 hour and 15 minutes.***

[If the interviewee is involved in **three** projects] *The interview will take about* ***1 hour and 30 minutes****.*

- - *Results will be* *implemented in* ***scientific publications.***
- Put the interviewee **at ease**:
  - *I want to emphasize that there are no right or wrong answers, and that it is no problem if you might not know the answer to a particular question. In that case, it would be great if you could recommend us the name of a contact person who could clarify this specific aspect.*
  - *This interview will be* ***digitally recorded****. This makes it easier for us to process all of the information that you provide us with during the interview. Anything you say today will be processed* ***pseudonymously****, meaning we will not use your name or any identifying information in publications following from this study. The data collected will be stored securely and viewed in unredacted form only by the researchers involved in this project.*
  - *Participation is* ***completely voluntary****. You can* ***withdraw*** *at any point, without having to give an explanation. You do not have to answer any questions during the interview if you do not feel comfortable answering them.*
- *Do you have any* ***questions*** *before we start the interview?*
- *We will start with some* ***warm-up questions****. Subsequently, we will turn on the* ***recorder*** *and focus more on the* ***research questions*** *which are organized across five main themes. The first theme concerns the project’s status and planning, the second theme will focus on EMA’s partnership with the external stakeholders, the third theme will deal with EMA’s input in the project, the fourth theme will address the learnings from the project and the fifth theme will cover some reflection questions.*

**Questions**

I. Introductory questions

1. Could you please start with shortly introducing yourself?
2. How long have you been working in your current position?
3. What are your expectations of this interview?

- *Let’s start with the research questions now. Do I have your permission to turn on the recorder?*

II. Questions about the project’s status and planning

1. What is the current status of the project?
2. What has been the impact of COVID-19 on the project?
3. To what extent is the project on track to realize its deliverables within the foreseen timeframe?

III. Questions about EMA’s partnership with the external stakeholders

1. How would you describe your relationship with the external stakeholders that are involved in this project?
2. How and how often do you communicate with these external stakeholders?
3. How has the scope of your interaction with the external stakeholders that are involved in this project changed since the start of the project?
4. In your view, what is working well in this project and partnership?
5. In your view, what is not working well in this project and partnership?

IV. Questions about EMA’s input in the project

1. What specific activities have you been involved in for this project? (e.g. agenda setting, document review, document writing, teleconferences, stakeholder liaison, ...)
2. According to your own estimates, how much time are you approximately spending on this project?
3. Which other EMA employees have been involved in this project, and what has been their contribution?
4. Which regulatory tools that the Agency has available to support external stakeholders in their activities have been used in this project so far? (e.g. scientific advice, Innovation Task Force, qualification advice/opinion, etc.)
5. To what extent do you think EMA participation in this project is still relevant at this point in time?
6. [If you think EMA participation is still relevant] What exactly can EMA still add according to you?

[If you think EMA participation is no longer relevant] What exactly has changed that makes EMA participation no longer relevant according to you?

V. Questions about the learnings from the project

1. In your view, what are the most important practical learnings for you and for EMA from this project so far?
2. In your view, what are the most important scientific learnings for EMA and for the wider EMA network from this project so far?
3. How have these scientific learnings been shared so far (e.g. publications, workshops, conferences)?

VI. Reflection questions

1. In your opinion, how does EMA's input in this project weigh against the outputs for the Agency?
2. Looking back with the knowledge that you have now about this project, what, if anything, would you have done differently?
3. How has your involvement in this project shaped the way in which you will approach similar projects in the future?
4. Is there any support you would need from the Academia Liaison Office that we are not offering at present?

VII. Round-up questions

1. Do you want to **add** anything else to what you’ve already said?
2. Do you want to **emphasize** something?
3. Do you think we **forgot or overlooked** some relevant questions?
4. Do you have any **questions for me**?

- *These were all the questions I had for you. I will turn off the recorder now.*

**Conclusion**

- ***Thank you*** *for your participation.*
- *If you have any other questions or comments, or if you want to get in touch with me, please do not hesitate to* ***contact me.***

***Interview guide EMA experts (finalized projects)***

**Introduction**

- **Present yourself**

*First, allow me to present myself. My name is Robbe Saesen and I am a PhD researcher at KU Leuven working at the Clinical Pharmacology and Pharmacotherapy research unit under the supervision of Professor Isabelle Huys. Within the context of the Collaborating Expert Programme, I am also working at the Task Force Regulatory Science & Innovation of the European Medicines Agency under the mentorship of Jordi Llinares Garcia.*

- **Thank** the interviewee for his/her participation in advance

*I would like to thank you already for taking the time to participate in this interview. Your views, opinions and experiences are very important to our research.*

- Explain **shortly the purpose** of the interview:
  - *With this interview we want to explore your experiences with participating in academia-coordinated regulatory science projects to which EMA has contributed* (keep it brief)
  - [If the interviewee is involved in **one** project] *The interview will take about* ***1 hour.***

[If the interviewee is involved in **two** projects] *The* *interview will take about* ***1 hour and 15 minutes.***

[If the interviewee is involved in **three** projects] *The interview will take about* ***1 hour and 30 minutes****.*

- - *Results will be* *implemented in* ***scientific publications.***
- Put the interviewee **at ease**:
  - *I want to emphasize that there are no right or wrong answers, and that it is no problem if you might not know the answer to a particular question. In that case, it would be great if you could recommend us the name of a contact person who could clarify this specific aspect.*
  - *This interview will be* ***digitally recorded****. This makes it easier for us to process all of the information that you provide us with during the interview. Anything you say today will be processed* ***pseudonymously****, meaning we will not use your name or any identifying information in publications following from this study. The data collected will be stored securely and viewed in unredacted form only by the researchers involved in this project.*
  - *Participation is* ***completely voluntary****. You can* ***withdraw*** *at any point, without having to give an explanation. You do not have to answer any questions during the interview if you do not feel comfortable answering them.*
- *Do you have any* ***questions*** *before we start the interview?*
- *We will start with some* ***warm-up questions****. Subsequently, we will turn on the* ***recorder*** *and focus more on the* ***research questions*** *which are organized across five main themes. The first theme concerns the project’s status and planning, the second theme will focus on EMA’s partnership with the external stakeholders, the third theme will deal with EMA’s input in the project, the fourth theme will address the learnings from the project and the fifth theme will cover some reflection questions.*

**Questions**

I. Introductory questions

1. Could you please start with shortly introducing yourself?
2. How long have you been working in your current position?
3. What are your expectations of this interview?

- *Let’s start with the research questions now. Do I have your permission to turn on the recorder?*

II. Questions about the project’s status and planning

1. What is the current status of the project?
2. What was the impact of COVID-19 on the project?
3. To what extent was the project able to realize its deliverables within the foreseen timeframe?

III. Questions about EMA’s partnership with the external stakeholders

1. How would you describe your relationship with the external stakeholders that were involved in this project?
2. How and how often did you communicate with these external stakeholders?
3. How did the scope of your interaction with the external stakeholders that are involved in this project change over the course of the project?
4. In your view, what worked well in this project and partnership?
5. In your view, what did not work well in this project and partnership?

IV. Questions about EMA’s input in the project

1. What specific activities were you involved in for this project? (e.g. agenda setting, document review, document writing, teleconferences, stakeholder liaison,...)
2. According to your own estimates, how much time did you approximately spend on this project?
3. Which other EMA employees were involved in this project, and what was their contribution?
4. Which regulatory tools that the Agency has available to support external stakeholders in their activities were used in this project? (e.g. scientific advice, Innovation Task Force, qualification advice/opinion, etc.)
5. Looking back now, to what extent do you think EMA participation in this project was relevant?
6. [If you think EMA participation was relevant] What exactly did EMA participation add to the project?

[If you think EMA participation was not relevant] What exactly changed from the initial proposal that in the end made EMA participation less relevant than expected?

V. Questions about the learnings from the project

1. In your view, what were the most important practical learnings for you and for EMA from this project?
2. In your view, what were the most important scientific learnings for EMA and for the wider EMA network from this project?
3. How were these scientific learnings shared (e.g. publications, workshops, conferences)?

VI. Reflection questions

1. In your opinion, how did EMA's input in this project weigh against the outputs for the Agency?
2. Looking back with the knowledge that you have now about this project, what, if anything, would you have done differently?
3. How has your involvement in this project shaped the way in which you will approach similar projects in the future?
4. Is there any support you would need from the Academia Liaison Office that we are not offering at present*?*

VII. Round-up questions

1. Do you want to **add** anything else to what you’ve already said?
2. Do you want to **emphasize** something?
3. Do you think we **forgot or overlooked** some relevant questions?
4. Do you have any **questions for me**?

- *These were all the questions I had for you. I will turn off the recorder now.*

**Conclusion**

- ***Thank you*** *for your participation.*
- *If you have any other questions or comments, or if you want to get in touch with me, please do not hesitate to* ***contact me.***
